# Improving the Performance of PbS Quantum Dot Solar Cells by Optimizing ZnO Window Layer

**DOI:** 10.1007/s40820-016-0124-2

**Published:** 2017-01-04

**Authors:** Xiaokun Yang, Long Hu, Hui Deng, Keke Qiao, Chao Hu, Zhiyong Liu, Shengjie Yuan, Jahangeer Khan, Dengbing Li, Jiang Tang, Haisheng Song, Chun Cheng

**Affiliations:** 1 Wuhan National Laboratory for Optoelectronics (WNLO) and School of Optical and Electronic Information, Huazhong University of Science and Technology, Wuhan, 430074 People’s Republic of China; 2 State Key Laboratory of Digital Manufacturing Equipment and Technology, Huazhong University of Science and Technology, Wuhan, 430074 People’s Republic of China; 3 Department of Materials Science and Engineering and Shenzhen Key Laboratory of Nanoimprint Technology, South University of Science and Technology, Shenzhen, 518055 People’s Republic of China

**Keywords:** ZnO, Window layer, Thin film solar cells, PbS quantum dots, Physical mechanism

## Abstract

Comparing with hot researches in absorber layer, window layer has attracted less attention in PbS quantum dot solar cells (QD SCs). Actually, the window layer plays a key role in exciton separation, charge drifting, and so on. Herein, ZnO window layer was systematically investigated for its roles in QD SCs performance. The physical mechanism of improved performance was also explored. It was found that the optimized ZnO films with appropriate thickness and doping concentration can balance the optical and electrical properties, and its energy band align well with the absorber layer for efficient charge extraction. Further characterizations demonstrated that the window layer optimization can help to reduce the surface defects, improve the heterojunction quality, as well as extend the depletion width. Compared with the control devices, the optimized devices have obtained an efficiency of 6.7% with an enhanced *V*_oc_ of 18%, *J*_sc_ of 21%, *FF* of 10%, and power conversion efficiency of 58%. The present work suggests a useful strategy to improve the device performance by optimizing the window layer besides the absorber layer.

**Electronic supplementary material:**

The online version of this article (doi:10.1007/s40820-016-0124-2) contains supplementary material, which is available to authorized users.

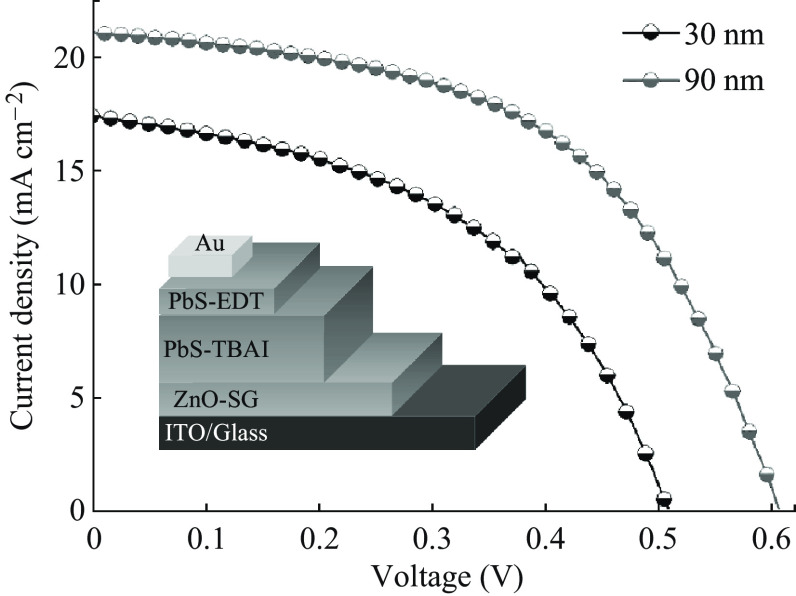

## Highlights

- The efficiencies of PbS solar cells was significantly improved from 4.3% to 6.7% by optimizing ZnO window layer.
- Optimized ZnO window layer can reduce the surface defects, extend thedepleted-heterojunction width and align with energy band of absorber layer.

## Introduction

Colloidal quantum dots (CQDs) have attracted significant attention for potentially wide applications in optoelectronic devices such as solar cells [1–3], photodetectors [4–6], and light-emitting diodes [7, 8] due to low-temperature fabrication, solution-based processing, and their peculiar optoelectronic properties [9–11]. For solar cell applications, the QDs’ bandgap can be conveniently tuned via the quantum size effect in order to match the wide absorption of solar spectra. Furthermore, recently the multi-exciton generation (MEG) effect in CQD-based solar cells (SCs) was reported, which can efficiently utilize high energy photons [1]. The above superior properties enable them as a promising light-absorbing material. In terms of device architecture, depleted-heterojunction ZnO–PbS SCs have achieved the state-of-art highest efficiency and demonstrated the outstanding atmosphere stability [12–14].

In depleted-heterojunction CQD SCs, there were numerous researches for optimizing absorber layers. In contrast, the window layer attracts less attention in spit that it plays the key roles in extracting and transporting charge carriers in heterojunction. As an n-type window layer, ZnO is an ideal candidate due to its relatively high electron mobility, environment stability, and high transparency [15]. Even utilizing the same window layer of ZnO, different groups utilized varied thickness and obtained over 8% conversion efficiency [12, 14, 16, 17]. Bawvendi et al. utilized 120 nm ZnO layer to achieve 8.5% certified efficiency [14]. Recently, Sargent group adopted 80 nm ZnO layer as n-layer and molecular-halide-passivated PbS QDs as absorber to obtain 9.9% certified efficiency [12]. Considering the optoelectronic function of the window layer, the varied thickness of ZnO layer needs further optimization for CQD SCs.

For ZnO layer fabrication, a sol–gel method was commonly used to prepare ZnO layer due to its low cost and simplicity [18–20]. However, the quality of solution-based ZnO film suffers from the surface defects or dangling bonds, which may act as charge trap sites or recombination centers [21–23]. To solve the above-mentioned problems, several strategies such as surface passivation or doping were reported to control the interfacial properties of heterojunction [24–28]. All of them have made promising progresses in the improvement of interface quality.

Herein, we adopted a layer-by-layer (LBL) sol–gel method to optimize the ZnO window layer. The modified sol–gel method could hold stronger capability to obtain smooth junction interface and finely control film processing. On the other hand, each layer deposition was followed one time of annealing. Thus, different ZnO layer thicknesses were corresponding to varied thermal treatment time as well as varied doping concentration [29, 30]. The performance of ZnO–PbS-QD solar cells was improved by optimizing ZnO window layer. The physical mechanism was also systematically investigated. Our work was expected to support an efficient routine for device performance improvement.

## Experimental Section

### Synthesis of PbS Quantum Dots

PbS CQDs were synthesized according to the modified literature method [31]. In this work, 0.9 g lead oxide (PbO, 99.9%) and 3 mL oleic acid (OA, 90%) were mixed with 20 mL 1-octadecene (ODE, 90%) in a 50-mL three-neck flask. The mixture was stirred and degassed at room temperature for 8 h and heated to 90 °C for 2 h. The obtained solution was then heated to 100 °C under nitrogen for 5 min, followed by injection of TMS (hexamethyldisilathiane (bis (trimethylsilyl) sulfide) solution (300 μL TMS mixed with 10 mL pre-degassed ODE) at 90 °C. After the reaction, the resulting solution was cooled to room temperature naturally. The obtained product was washed and purified 4 times by dispersion/precipitation in hexane/acetone. Finally, the cleaned QDs were dispersed in hexane and octane (vol:vol = 4:1) mixed solvents with ~15 mg mL^−1^ to be ready for use.

### Layer-by-Layer Sol–Gel Method Deposition of ZnO Film

The ZnO precursor was prepared by dissolving 1.5 g zinc acetate dehydrate (Zn(Ac)_2_·2H_2_O, sinopharm, 99%) and 400 μL ethanolamine (NH_2_CH_2_CH_2_OH, sinopharm, 99%) in 20 mL 2-methoxyethanol (CH_3_OCH_2_CH_2_OH, sinopharm, 99%) under vigorous stirring at 60 °C for 10 h for the hydrolysis reaction in air. On a precleaned ITO/glass substrate, ZnO precursor solution was spin-coated at 4000 r min^−1^ for 30 s and annealed at 400 °C for 15 min, followed by repeating this process some times to reach the required thickness.

### Device Fabrication

PbS CQD films were fabricated by layer-by-layer spin-coating according to the published reports [14]. For tetrabutylammonium iodide (TBAI) ligand exchange process, QDs dispersed in hexane/octane mixed solvents was dropped on ZnO-coated substrate and then immediately spinned at 2500 r min^−1^ for 10 s. The obtained film was soaked in TBAI (10 mg mL^−1^ in methanol) solution for 1 min, followed by two-time methanol rinsing. This process obtained a TBAI-treated QD layer and the number of layers was 10–12. For PbS-EDT (1,2-ethanedithiol) layer, 0.01 vol% EDT/acetonitrile solution was used and spinned after 30 s soaking, which was followed by a 3-time acetonitrile rinsing. This process was repeated two times. The total thickness of PbS CQD film was ~240 nm. Finally, 100 nm Au was evaporated on PbS film to complete the device fabrication. The active device area (9 mm^2^) was defined by shadow mask. It is noted that majority of high-efficiency PbS QDSCs reported so far were obtained based on small area (<5 mm^2^) which was almost half of our device area.

### Characterizations

The ZnO films were investigated by X-ray diffraction (XRD) with Cu Ka radiation (Philips, X pert pro MRD, Netherlands), UV–Vis absorption spectra (Cary, Lambda 950, America), Hall effect (Ecopia, HMS-5500, Korea), photoluminescence (PL, LabRAM HR800, France), and X-ray photoelectron spectroscopy (XPS, EDAX Inc. Genesis, America). The device cross-section was obtained from using scanning electron microscopy (FEI Nova 450, America). The *J*–*V* characteristics were measured by a Keithley 2400 source unit with Xenon lamp (Newport, 3A solar simulator, 94023A-U, Germany) as the light source with simulated air mass (AM) 1.5G irradiation at 100 mW cm^−2^. The external quantum efficiency (EQE) measurements were taken by a home-made setup containing a Keithley 2400 Source Measure unit and Newport monochromator. The output power was also calibrated by Si photodetectors. The work function of various ZnO films was measured by using a Scanning Kelvin Probe microscopy (SKPM, UHV-KP, KP technology, Britain) in air at dark condition. The *C*–*V* measurements were acquired with an Agilent 4200A at a frequency of 10 kHz and AC signal of 50 mV, scanning from −1 to +0.6 V, with a step size of 50 mV. The EIS of the QD SCs was performed on an electrochemical workstation (Autolab PGTSAT302N, Metrohm Autolab, Utrecht, Netherlands) in the dark with the frequency ranging from 0.1 to 10^6^ Hz.

## Results and Discussion

**Fig. 1:**
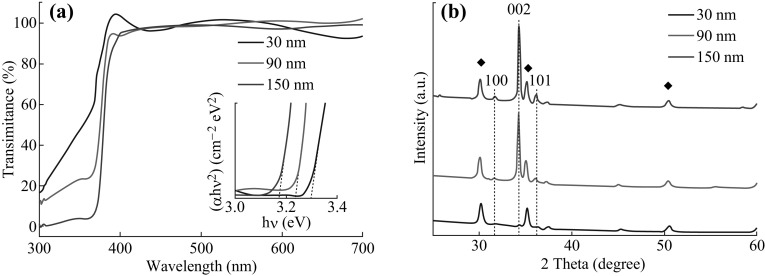
**a** Transmittance spectra (*Inset*: the Tauc plots of various thickness ZnO layers). **b** XRD patterns of ZnO films on ITO/glass substrate. ITO peaks are marked by black diamonds and ZnO peaks are identified by their Miller indices

As a window layer, the optical transmittance determined the light response of absorber layer in solar cells. Considering the varied window layer thickness effect, three typical thicknesses of 30, 90, and 150 nm were prepared to investigate the thickness-dependent optoelectronic properties. Figure 1a shows the UV–Vis transmittance spectra of three typical ZnO layers. As the ZnO film thickness increases, the onset absorption is red shift. The optical band gaps (*E*_g_) extracted from Tauc plots [32] (inset of Fig. 1a) are 3.35, 3.26, and 3.18 eV, respectively. From the onset of absorption spectra, there are tail states extending into the bandgap (inset), which may have arisen from impurities and defects at grain boundaries [33–35]. Interestingly, the increased thickness would increase the losses of light absorption, while it simultaneously reduces the densities of tail states, which would be explained in the latter part by different thermal treatment times. Therefore, it is necessary to optimize the ZnO film thickness to balance the transmittance and the density of tail states. XRD patterns (Fig. 1b) for different thicknesses of ZnO films indicate that the crystallinity with wurtzite structure could be enhanced as the thickness increased [15, 36]. Moreover, the *c*-axis oriented (002) intensity of thicker ZnO film is stronger than the thinner ones, demonstrating the orientation growth which may improve the carrier transport mobility [37].

The thickness-dependent electrical properties were characterized by Hall measurement (Fig. S1b) or field-effect transistors (FET) [38] (Fig. S1c–d). It is worth noting that electrical properties of the thinner ZnO (30 nm) film was too insulating to be tested by Hall measurement, and thus we converted to FET testing. The extracted electrical results are listed in Table 1. The carrier mobility of thicker ZnO films increases one order more than the thinner ones. And the corresponding carrier concentrations increase by two orders as the film thickness increased. The higher carrier mobility of thick film can be explained with the decreased concentration of grain boundaries in thicker films [39].

**Table 1:** Thickness-dependent electrical properties for varied thickness of ZnO layer

Thickness of ZnO film (nm)	Carrier concentration (cm^−3^)	Mobility (cm^2^ v^−1^ s^−1^)	Conductivity (S cm^−1^)
30	1.05 × 10^16^	8.7 × 10^−3 a^	2.25 × 10^−3^
90	1.02 × 10^18^	3.64 × 10^−1 b^	5.92 × 10^−2^
150	1.70 × 10^18^	1.04 × 10^−1 b^	2.83 × 10^−2^

^a^The values are extracted from FET measurements

^b^The data are obtained from Hall measurements

**Fig. 2:**
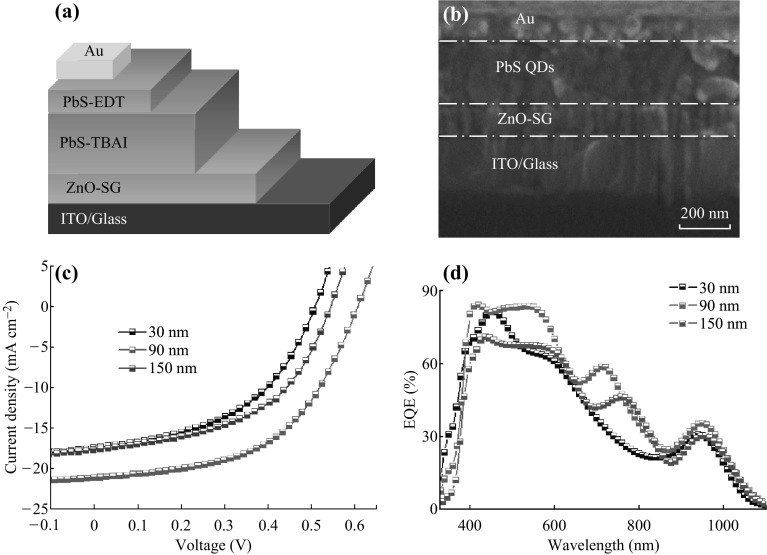
**a** Schematic device structure. **b** Cross-section SEM image of ZnO–PbS QD device. **c** Representative *J*–*V* characteristics. **d** *EQE* curves of devices with various ZnO film thicknesses

Figure 2a shows the schematic device structure of ZnO–PbS QD SCs consisting of n-type ZnO layer and p-type PbS QD absorber layer. The bandgap of QDs used in this work is 1.39 eV (Fig. S1a). The thickness of ZnO and PbS films was strictly confirmed by scanning electron microscopy (SEM) characterization. Figure 2b shows the sharp contrast from different functional layers in cross-section image.

**Fig. 3:**
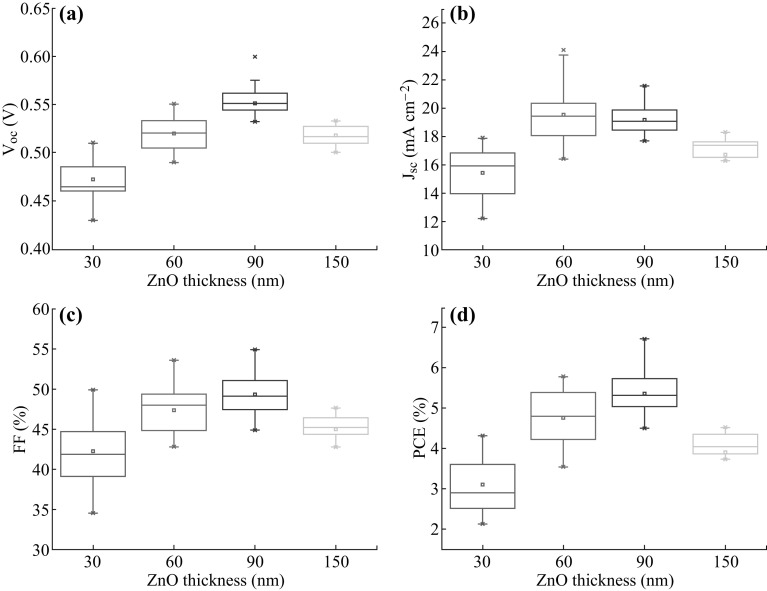
Statistics of device performance using various thicknesses of ZnO window layer (*x* = 30, 60, 90, and 150 nm): **a** *V*_oc_, **b** *J*_sc_, **c** *FF*, **d** *PCE*. The *solid squares* represent the average values, and the *horizontal lines in the box* denote the 25th, 50th, and 75th percentile values

The *J*–*V* characteristics of ZnO–PbS QD SCs with three representative thicknesses (30, 90, and 150 nm) are shown in Fig. 2c, where the corresponding ZnO film layers are denoted as C-ZnO (control ZnO layer, 30 nm), O-ZnO (optimized ZnO layer, 90 nm), and T-ZnO (thicker ZnO layer, 150 nm). The control devices with C-ZnO exhibit a *J*_sc_ of 17.42 mA cm^−2^, a *V*_oc_ of 0.51 V, and a *FF* of 48%, leading to a *PCE* of 4.26%. According to the Site’s method [40], the series resistance (*R*_S_) and shunt resistance (*R*_sh_) are 7.6 and 160.56 Ω cm^2^, respectively. As the ZnO layer thickness increases, the *PCE* of CQD SCs is firstly increased and the champion device (O-ZnO) reaches 6.7% with a *J*_sc_ of 21.08 mA cm^−2^ when the thickness of ZnO films is 90 nm. Further increase would lead to the deterioration of *PCE*. The detailed ZnO film and device parameters are summarized in Table 2. From the comparison between them, all the parameters of O-ZnO are simultaneously improved. In order to guarantee the credible device performance, sixteen to twenty devices of each type were fabricated and their parameter distribution is shown in Fig. 3. Their statistical deviations are small, and thus our improvement is reliable.

**Table 2:** Device performance parameters obtained from Fig. 2c

Device	*μ* (cm^2^ (v s)^−1^)	*V*_oc_ (V)	*J*_sc_ (mA cm^−2^)	FF (%)	*η* (%)	*R*_s_ (Ω cm^2^)	*R*_sh_ (Ω cm^2^)	*J*_0_ (mA cm^−2^)
30-nm (C-ZnO) SCs	8.7^ a^	0.51	17.42	47.89	4.26	7.6	160.58	1.3 × 10^−3^
90-nm (O-ZnO) SCs	3.64 × 10^−1 b^	0.60	21.08	52.79	6.73	2.6	273.2	1.4 × 10^−4^
150-nm (T-ZnO) SCs	1.04 × 10^−1 b^	0.54	17.63	49.87	4.78	8.8	151.47	7.8 × 10^−4^

^a^The values are extracted from FET measurements

^b^The data are obtained from Hall measurements

To study the origin of *J*_sc_ improvement, EQE spectra of three types of ZnO film-based devices are shown in Fig. 2d. There are three characteristic regions from *EQE* comparison. In ultra-violet region (300–400 nm), the response in control devices is highest, which agreed well with aforementioned absorption measurement results of ZnO films. Thus, the response loss for devices based on O-ZnO and T-ZnO film is mainly caused by window layer absorption. In visible region (500–800 nm), O-ZnO devices demonstrate higher and broader response. This result demonstrates that O-ZnO devices could more efficiently extract electrons from PbS QD layers. In infrared region, all three *EQE* values are similar among these devices, which confirm the efficient back field in PbS-TBAI/PbS-EDT device structure [14]. To investigate the contribution of *J*_sc_ for various ZnO–PbS devices, Fig. S3a shows the integrated short-currents for C-ZnO and O-ZnO devices. Compared with C-ZnO film devices, although a loss of light absorption is found in first region (Region I, UV spectrum) for O-ZnO device, the more contributions of short-currents can be obtained from second and third regions (Region II and III, visible and infrared regions). The current density variations corresponding to Region I–III are 0.25, 2.21, and 1.09 mA cm^−2^, respectively. Consequently, the O-ZnO devices could more efficiently convert visible and infrared spectra into photocurrent. On the other hand, UV spectra energy only takes 4% while the visible and infrared spectra energy takes more than 90% in solar spectra energy distribution. Based on the above analysis, the improved quantum efficiency in visible and infrared regions is the main contribution to achieve the higher short-currents of ZnO–PbS SCs.

**Fig. 4:**
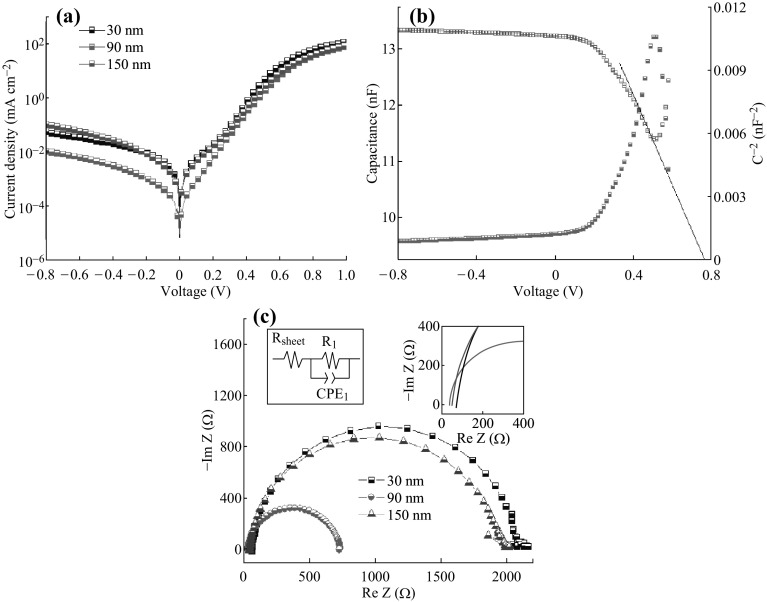
**a** Logarithmic plots of dark *J*–*V* characteristics. **b** Capacitance–voltage measurement results and Mott–Schottky plots of ZnO–PbS QD SC-based O-ZnO, the red and blue curves are represented *C*–*V* and C^−2^-V evolutions, respectively. **c** The AC impedance spectroscopy of the QD SCs with various ZnO films. *Insets* indicate the equivalent circuit model (*left*) and intercept (*right*) with the horizontal axis, respectively

In order to gain the physical origins of the improved parameters (*V*_oc_, *J*_sc_, *FF*) in O-ZnO device, further characterizations were carried out for various thicknesses of ZnO film and their corresponding devices. Figure 4a shows the logarithmic plots of dark *J*–*V* curves. The reverse saturation current is greatly suppressed in O-ZnO devices. The lower *J*_0_ value (2.18 × 10^−4^ mA cm^−2^) demonstrates the superior heterojunction quality which also can be confirmed from the higher *R*_sh_ in Fig. S2b. It is known that *V*_oc_ depends strongly on the ideal factor (*n*) and reversed saturation current density (*J*_0_), described by Eq. 1 [41].

1 $$ V_{\text{OC}} = \frac{nKT}{q}\left( {\ln \frac{{J_{\text{SC}} }}{{J_{0} }} + 1} \right), $$

where *q* is the elementary charge. According to Eq. 1, O-ZnO device has higher *V*_oc_ than others due to its lower reverse saturation current and higher *J*_sc_. For the heterojunction analysis, the work function (*W*_f_) of three kinds of window layers was measured from the SKPM results (Fig. S3b). With the increase of ZnO film thickness, the *W*_f_ became shallower which could help to improve the *V*_oc_ of corresponding devices [42].

Because of the higher *J*_sc_, we further analyzed the depletion region of ZnO/PbS QD SCs by capacitance versus voltage (*C*–*V*) measurements as shown in Fig. 4b and S3d. The built-in voltage (*V*_bi_) is extracted from the intercept of 1/C^2^ curve on horizontal axis. The dopant concentration (*N*_A_) for PbS QD layer could be extracted from the slope of the Mott–Schottky plot from Eq. 2 [43, 44],

2 $$ N_{\text{A}} = \frac{2}{{A^{2} q\varepsilon_{\text{QD}} \varepsilon_{0} \frac{\text{d}}{{{\text{d}}V}}\left( {\frac{1}{{C^{2} }}} \right)}}, $$

where *ε*_0_ is the dielectric constant of vacuum, *ε*_QD_ is the QD dielectric constant extracted as shown in Fig. S3c, and *A* corresponds to the device active area. Substituting all the parameters in Eq. 2, the carrier concentration of PbS QD layer is extracted as 4.79 × 10^16^ cm^−3^, which is in accordance with reported values of TBAI-treated PbS QD films [12]. Utilizing the above carrier concentration, we can calculate the depleted width of QD layer (*W*_PbS_) at zero bias according to Eq. 3 [44].

3 $$ W_{\text{PbS}} = \frac{1}{{N_{\text{A}} }}\left[ {\frac{{2\varepsilon_{\text{QD}} \varepsilon_{0} }}{{q\left( {\frac{1}{{N_{\text{D}} }} + \frac{1}{{N_{\text{A}} }}} \right)}}V_{\text{bi}} } \right]^{{\frac{1}{2}}}, $$

where *N*_D_ is the carrier concentration of ZnO film, which is extracted by FET or Hall measurements as mentioned above (Table 1). The obtained parameters are summarized in Table 3. The carrier concentration evolution with varied thickness is mainly attributed to our LBL sol–gel method. The thicker ZnO film faces longer thermal treatment time, inducing varied doping concentration [45, 46]. It is noteworthy that Mott–Schottky analysis in a Schottky junction or an abrupt heterojunction must be based on the premise, in which the carrier concentration of N-type layer must be much higher than that of p-type layer [47]. Therefore, the C-ZnO-based parameters could not meet the Mott–Schottky equation. Here we roughly estimate *W*_PbS_ according to the device based on 60 nm ZnO film (Table S1) and Eq. 4 [48],

4 $$ N_{\text{D}} \times W_{\text{ZnO}} = N_{\text{A}} \times W_{\text{PbS}} $$

**Table 3:** Device performance parameters extracted from the Mott–Schottky analysis

Devices	*V*_bi_ (V)	*N*_D_ (cm^−3^)	*N*_A_ (cm^−3^)	*W*_D, ZnO_ (nm)	*W*_PbS_ (nm)
C-ZnO–PbS	–	1.6 × 10^16^	~4 × 10^16^	≤30	<151
O-ZnO–PbS	0.79	1.0 × 10^18^	4.8 × 10^16^	8.8	185
T-ZnO–PbS	0.76	1.7 × 10^18^	4.5 × 10^16^	5.1	190

The *W*_PbS_ for C-ZnO-based devices (<151 nm, referring to Table S1) is much narrower than the other two devices. The *W*_PbS_ for O-ZnO-based devices extends to 185 nm (Table 3). The higher carrier concentration of ZnO films could help to extend the *W*_PbS_ and enhance the electrical field resulting in the improvement of the charge-collection efficiency.

For *FF* enhancement analysis, EIS was measured to investigate the interfacial properties. Figure 4c shows the Nyquist plots of varied thickness of ZnO film-based devices. Only one semicircle is obtained in these devices regardless of the ZnO film thickness. From their equivalent circuit diagrams and intercept with the horizontal axis, the O-ZnO-based devices extract a smaller series resistance. Thus, the higher *FF* in O-ZnO PbS QDSCs is ascribed to the decreased *R*_s_ [26].

**Fig. 5:**
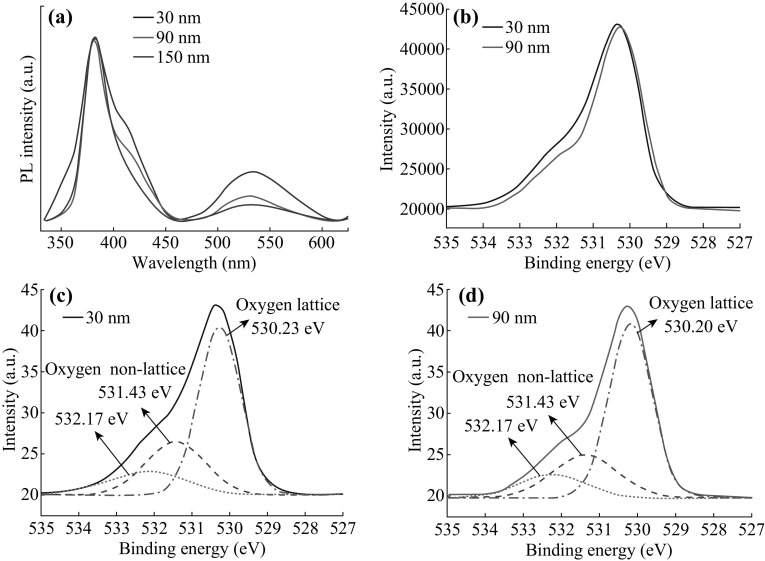
**a** Steady-state normalized PL spectra of various ZnO layers excited at 325 nm. **b** XPS survey spectra of the O1s core level of C-ZnO film (30 nm) and O-ZnO (90 nm). Deconvoluted XPS spectra of **c** C-ZnO, and **d** O-ZnO

In addition, the window layer ZnO film itself also plays the key role in device performance. Photoluminescence (PL) spectra and X-ray photoelectron spectroscopy (XPS) reveal more details for its functionality. As shown in Fig. 5a, there are approximately two emission peaks in PL spectra. One is centered at ~365 nm corresponding to band-edge emission, and the other broad one located at ~530 nm is attributed to the oxygen vacancy (*V*_O_) defect-related emission [49–51]. It is clearly shown that the visible emission is strongly suppressed in thicker film. Thus, the rich defects in C-ZnO-based devices may lead to the *EQE* losses within 500–600 nm region corresponding to the defect absorption [49]. As mentioned above, the depletion region is mainly located at PbS layers because ZnO has a higher carrier concentration (10^18^ cm^−3^) than that of PbS-CQDs (10^16^). With the help of built-in electric field in the depletion region, the separated carriers can be more efficient drift and collection than diffusion region. Thus, the effective passivation by annealing in thick ZnO film may be the crucial origin of the reduced charge recombination [52].

The XPS spectra provide more details in terms of the surface component of ZnO. The O1s core level spectra of the C-ZnO and O-ZnO are shown in Fig. 5b. In general, the peak for ZnO are deconvoluted into three peaks: the lower-binding-energy peak (530.2 eV) is associated with the oxygen atoms in a ZnO matrix, the higher-binding-energy peaks (532.17 and 531.43 eV) are attributed to the oxygen-deficient defects such as oxygen vacancies and hydroxyl OH groups (Fig. 5c, d) [37]. After increasing the thickness (annealing time), the relative intensities of higher-binding-energy components decreased (Fig. 5b, d), suggesting that the oxygen-deficient defects in the ZnO films are suppressed. These results, together with PL analyses, indicate that the thicker film could help to passivate window layer defects.

**Fig. 6:**
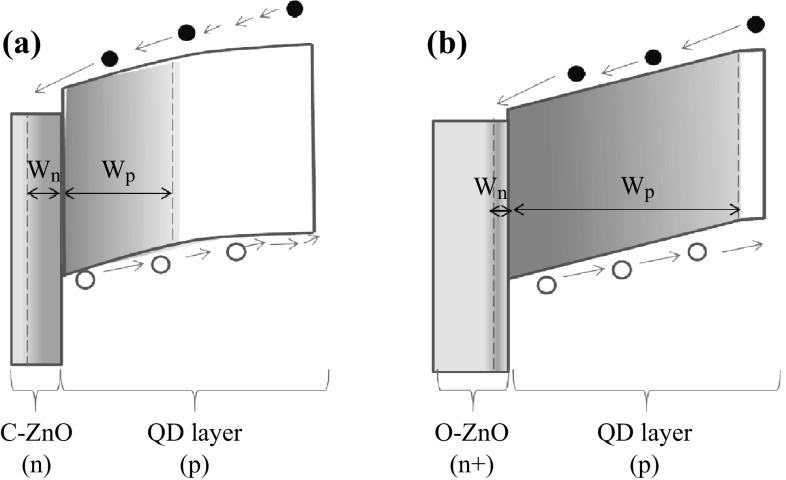
Schematic depletion region evolution as the effect of ZnO layer. **a** C-ZnO–PbS heterojunction, and **b** O-ZnO–PbS heterojunction QDSCs. *W*_n_ and *W*_p_ are the depletion region widths of window and absorber layers, respectively

In consequence, the appropriate thickness of ZnO films and suitable annealing time suppressed the interfacial charge recombination at the ZnO–PbS interface to enhance the charge separation at heterojunctions. It could also improve the ZnO films dopant concentration, which may be caused by the interstitial Zn_I_ rather than V_O_ [53]. The heavier doping of ZnO layer could help to extend depletion width in QD layer leading to a broader *EQE*. Thus, charge extraction properties of C-ZnO and O-ZnO devices can be schematically described as shown in Fig. 6a, b. *W*_n_ and *W*_p_ represent the depletion region widths in the ZnO layer and QD layer, respectively. The increased doping concentration of O-ZnO could extend the *W*_p_, suppress the recombination, and improve the short-current density.

## Conclusions

In the present work, we have successfully demonstrated an obvious improvement in the performance of ZnO–PbS QD SCs via optimizing the window layer. The optimized O-ZnO window layer-based PbS QD SCs showed an enhanced *PCE* of 58% compared with control devices. The physical mechanism for enhanced parameters (*V*_oc_, *J*_sc_, and *FF*) was also systematically illustrated. It demonstrated that the O-ZnO could reduce the surface defects, extend the depleted width in heterojunction, and align with energy band of absorber layer. The above effects could be conveniently implemented by optimizing the ZnO film thickness and its parasitic thermal treatment. The present simple and reliable optimizing strategy may provide a viable reference for depleted-heterojunction solar cells.

## Electronic supplementary material

Below is the link to the electronic supplementary material.

Supplementary material 1 (PDF 711 kb)
