# Supplementary material for: Improving the Performance of PbS Quantum Dot Solar Cells by Optimizing ZnO Window Layer
Source: Nanomicro Lett. 2017 Jan 4;9(2):24. doi: 10.1007/s40820-016-0124-2 (PMC6223798; doi:10.1007/s40820-016-0124-2)
Supplement: Supplementary file 1 — Supplementary material 1 (PDF 711 kb) [file 40820_2016_124_MOESM1_ESM.pdf]

Supporting Information for

Improving the Performance of PbS Quantum Dot Solar Cells by Optimizing ZnO Window Layer

Xiaokun Yang[1,3], Long Hu[1], Hui Deng[1], Keke Qiao[1], Chao Hu[1], Zhiyong Liu[2], Shengjie Yuan[1], Jahangeer Khan[1], Dengbing Li[1], Jiang Tang[1], Chun Cheng[3,]*, Haisheng Song[1,]*

[1]Wuhan National Laboratory for Optoelectronics (WNLO) and School of Optical and Electronic Information, Huazhong University of Science and Technology, Wuhan 430074, People's Republic of China

[2]State Key Laboratory of Digital Manufacturing Equipment and Technology, Huazhong University of Science and Technology, Wuhan 430074, People's Republic of China

[3]Department of Materials Science and Engineering and Shenzhen Key Laboratory of Nanoimprint Technology,South University of Science and Technology, Shenzhen 518055, People's Republic of China

*Corresponding author.

Email: cheng.c@sustc.edu.cn, songhs-wnlo@mail.hust.edu.cn

1 Calculation of Carrier Concentration of Varied Thickness of ZnO Film

The carrier concentration of O-ZnO and T-ZnO can be measured by Hall-effect measurement. However, the carrier concentration of C-ZnO can not be obtained from the same way, but from the fied-effect-transistor device characterization. In this method, the field-effect mobility of the ZnO layer was calculated from the two following formulas [1]:

$$\mu = \frac{dI_{DS}}{dV_{GS}} \cdot \frac{L}{WC_{SiO_2}V_{DS}} \qquad (1)$$

where L, W, and C_{SiO_2} are the channel length, channel width, and the capacitance for SiO_2, respectively.

$$\rho = n\, q\, \mu \qquad (2)$$

where ρ is the conductivity, q is the elementary charge, and n is the carrier concentration.

2 Scanning Kelvin Probe Microscopy Measurement

The V_{oc} enhancement was further strengthened from their energy band alignement. The surface potential shift of ZnO film was measured by scanning Kelvin probe microscopy (SKPM). As shown in Fig. S2b, the surface potential evolved from -4.35 eV for C-ZnO to -4.28 eV for O-ZnO films, which closed to conduction of PbS-TBAI layer [2] and led to the corresponding change of V_{oc}. The V_{oc} evolution trend agreed well with the device statistical results in Fig. 3. And the highest V_{oc} ~0.60 V was obtained from the O-ZnO film based devices.

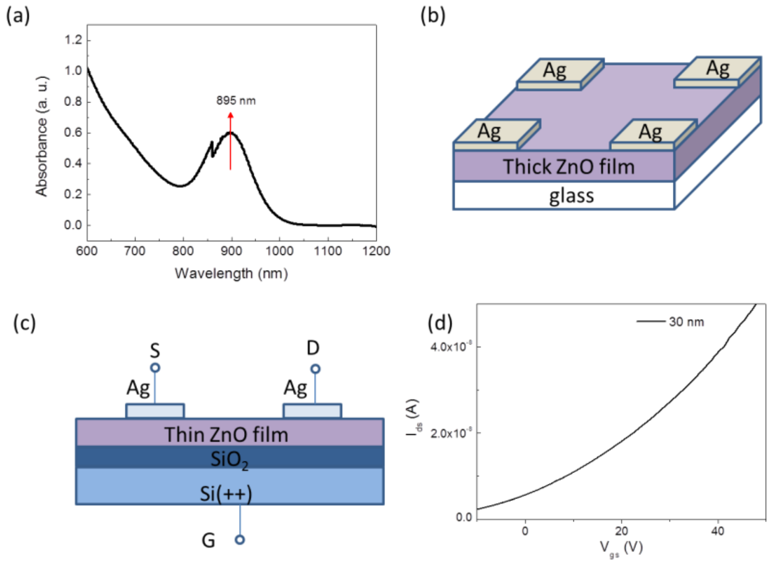

Fig. S1 a Absorption spectra of PbS QDs in hexane solution. The device structure used for the **b** Hall effect measurement and **c** FET measurement. **d** Transfer characteristics of C-ZnO FET

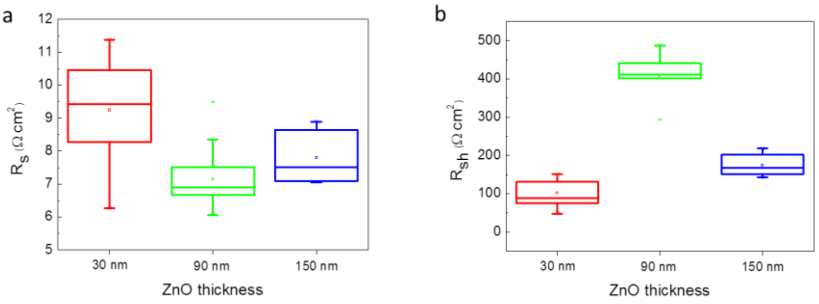

Fig. S2 Statistics of device **a** series resistances and **b** shunt resistances using varied thickness of ZnO window layer

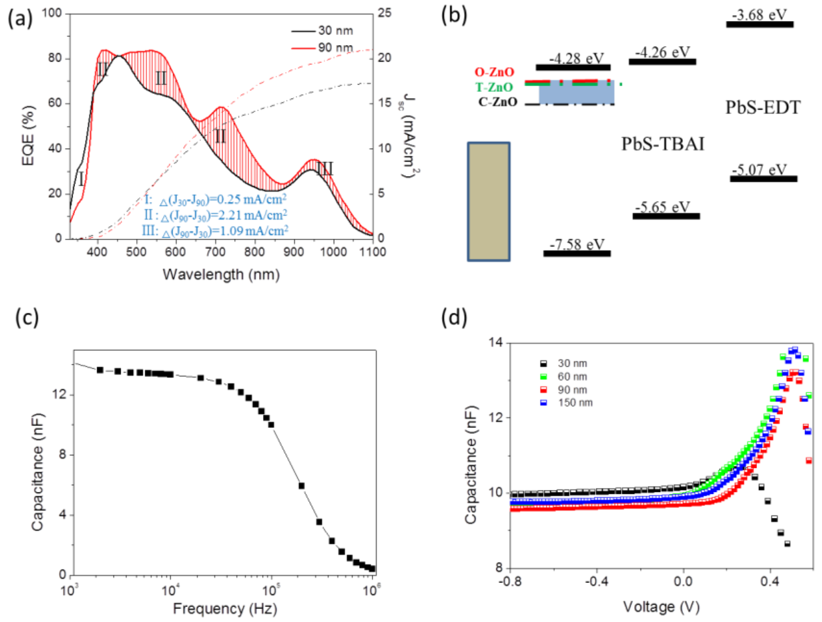

Fig. S3 a *EQE* and integral current curves for 30 and 90 nm-ZnO devices and corresponding current contribution difference in different spectra regione. **b** The schematic energy level diagram for the ZnO-PbS QDs devices. **c** *C-F* measurement data of ITO/PbS/Au ranging from 10^3 to 10^6 Hz. By getting the *C* value at 1~10 KHz, then ε_{QD} can be obtained from the following formula: $C = \frac{\varepsilon_r \varepsilon_0 S}{d}$, where *S* is active area and *d* is the thickness of PbS-TBAI layer. **d** *C-V* measurements data of ZnO-PbS QDSCs with various thickness of ZnO films

Table S1 The Mott-Schttky analysis of the 60 nm-ZnO devices

**Devieces**	**V_{bi} (V)**	**N_D (cm^{-3})**	**N_A (cm^{-3})**	**$W_{D, ZnO}$ (nm)**	**W_{PbS} (nm)**
**60 nm-ZnO-PbS**	0.70	2.9×10^{17}	4.57×10^{16}	23.7	151.2

References

[1] D. Yang, B. Li, C. Hu, H. Deng, D. Dong et al., Controllable growth orientation of SnS_2 flakes for low-noise, high-photoswitching ratio and ultrafast phototransistors. Adv. Opt. Mater. **4**, 419-426 (2016). doi:10.1002/adom.201500506

[2] C.H. Chuang, P.R. Brown, V. Bulovic, M.G. Bawendi, Improved performance and stability in quantum dot solar cells through band alignment engineering. Nat. Mater. **13**(8), 796-801 (2014). doi:10.1038/nmat3984
